# Evaluation of the Power Up program: a health promotion program encouraging healthy lifestyle habits among youth in summer day camps—study protocol

**DOI:** 10.3389/fpubh.2025.1521438

**Published:** 2025-02-26

**Authors:** David Larose, Melvin Chih-Shing Chen, Tania Paracini, Shirin Panahi, Jennifer Yessis, Angelo Tremblay, Vicky Drapeau

**Affiliations:** ^1^Department of Kinesiology, Université Laval, Québec, QC, Canada; ^2^Quebec Heart and Lung Institute Research Center, Université Laval, Québec, QC, Canada; ^3^Centre Nutrition, santé et société (NUTRISS), Institute of Nutrition and Functional Foods (INAF), Université Laval, Québec, QC, Canada; ^4^Centre de recherche interuniversitaire sur la formation et la profession enseignante (CRIFPE), Université Laval, Québec, QC, Canada; ^5^School of Public Health Sciences, University of Waterloo, Waterloo, ON, Canada; ^6^Fondation Tremplin Santé, Québec, QC, Canada

**Keywords:** summer day camps, physical activity, sedentary behaviors, healthy eating, campers, counselors

## Abstract

**Introduction:**

During the summer holidays, children often demonstrate reduced physical activity and poorer dietary habits, largely attributed to the lack of structured routines and supervision that school provides. Summer camps have the potential to offer youth engaging and organized activities and serve as an environment for promoting healthy lifestyle habits. This paper presents the protocol for the evaluation of the Power Up program, a study which aims to evaluate counselors' satisfaction with the Power Up services, trainings, and tools, their engagement in the program, as well as their self-efficacy and intention to promote physical activity, nutrition, and well-being through the camp environment. The secondary aim is to assess the program's effectiveness on physical activity, sedentary behaviors, and healthy eating among counselors and campers, along with the determinants of these behaviors.

**Methods:**

This quasi-experimental study will evaluate the efficacy of implementing Power Up, a healthy lifestyle promotion program in summer camps. Power Up offers a range of services, trainings, and tools designed to assist camps in promoting healthy lifestyle habits, all developed based on the Social Cognitive Theory. Camps can choose from various services based on their needs, including personalized support, funding, challenge-based activities and contests. Training for camp counselors is provided with additional advanced workshops and webinars available. The program also includes over 150 tools to promote healthy lifestyle habits, such as physical activity, sedentary behaviors, and healthy eating. This study plans to enroll counselors aged 15 to 21 and campers aged 8 to 12 in two Canadian provinces from multiple summer camps over 3 years. Due to constraints that complicate the inclusion of a control group, an implementation score will be used to document the program's effects based on its degree of implementation in the camps. Descriptive statistics and MIXED linear models for repeated measures will be used to assess the effects of time, group (high vs. low implementation) and their interactions on behaviors and their determinants.

**Conclusions:**

The results will provide evidence regarding the effectiveness of implementing Power Up to promote the adoption of healthy lifestyle habits among counselors and campers and its effect on the camp environment.

## Introduction

A healthy lifestyle, including regular physical activity and healthy eating habits during childhood and adolescence, is linked to optimal physical, cognitive, and socio-emotional development (1). However, youth generally do not maintain these optimal lifestyle behaviors, with few being active and consuming fruits and vegetables daily (2, 3). These behaviors tend to deteriorate further during the summer months, especially when children have less structured routines, limited access to organized activities, and decreased supervision, which leads to reduced physical activity and unhealthy dietary habits (4, 5). This is particularly true for youth from low socioeconomic backgrounds, who face additional barriers such as limited access to recreational spaces and resources, exacerbating the risk of increased obesity rates and decreased physical fitness (6, 7).

Summer camps may play a vital role in addressing these challenges as they represent an ideal environment that can foster motivation and encouragement to be active and provide access to equipment and facilities (8). Moreover, with trained counselors and structured programs, summer camps offer a supervised environment where healthy lifestyle habits can be promoted among campers (9). Recognizing the gaps in children's lifestyle habits and the conducive environments for promoting healthy behaviors in camps, Power Up was established to assist camps and their staff through various initiatives, including training sessions, tools, and challenge-based activities and contests.

This paper presents the protocol for a study evaluating the Power Up program. The primary aim of this study is to evaluate the counselors' satisfaction with the Power Up program's services, trainings, and tools, their engagement in the program, along with their self-efficacy and intention to promote physical activity, nutrition, and wellbeing within the camp environment. The secondary aim of the study is to assess the program's effectiveness on counselors' and children's physical activity, sedentary behaviors, and healthy eating, as well as the determinants of these behaviors. The hypothesis is that camps with strong implementation of the Power Up intervention will show sustained or notable improvements in physical activity, sedentary behaviors, and healthy eating among counselors and campers, driven by counselors' satisfaction, improved self-efficacy, and intention to use the provided resources and support.

## Methods

### Study design

The study will use a quasi-experimental design to evaluate the Power Up program, a health promotion initiative targeting camps with low socio-economic status, implemented across 1,054 summer camps in Canada. Data will be collected annually over a 3-year period in two provinces: Ontario (University of Waterloo) and Quebec (Université Laval). The primary outcomes include counselors' satisfaction, self-efficacy, and intention, which will be measured after they receive Power Up training before the camps. Additionally, counselors' engagement in the program, particularly through the use of provided tools and resources, will be assessed at the end of the summer to evaluate how effectively they implemented these tools in their activities. Lifestyle behaviors (i.e., physical activity, sedentary behaviors, and healthy eating) and their determinants will be assessed in both counselors and campers at the beginning and end of the camp. This design allows for a comprehensive evaluation of the program's effectiveness across diverse settings and populations (10, 11).

### Ethics and dissemination

The research project underwent ethical review by the University of Waterloo and Université Laval institutional review boards. The ethics board at the University of Waterloo approved the project, requiring that participants (counselors and campers) from Ontario be included through passive consent. Conversely, Université Laval's ethics board determined that this project qualifies as an exempt study under TCPS 2.8 (10), as it is considered a program evaluation activity. Participants and parents will be informed about the evaluation by email and newsletters.

### Recruitment

This study will use a convenience sampling method, selecting participants based on their availability and willingness to participate rather than through random sampling. The evaluation will recruit campers aged 8 to 12 and counselors aged 15 to 21 working in summer day camps participating in the Power Up program. Camp recruitment will be conducted by the research team in collaboration with Power Up program's management team. A call for participation will initially be made and then camps will be chosen based on geographical criteria to facilitate data collection. Three camps per province will be recruited each year over 3 years. Camps not engaged in the Power Up program or those focused on specific activities, such as sports or arts, will be excluded from the evaluation.

Campers will not receive compensation for participating in evaluation activities conducted at camp. However, counselors who completed the training evaluation questionnaire and the healthy lifestyle questionnaires at both the start and end of the project will receive a $20 incentive. Additionally, participating camps will be awarded $300 to acknowledge the time devoted to the evaluation.

### Intervention

**Figure 1:**
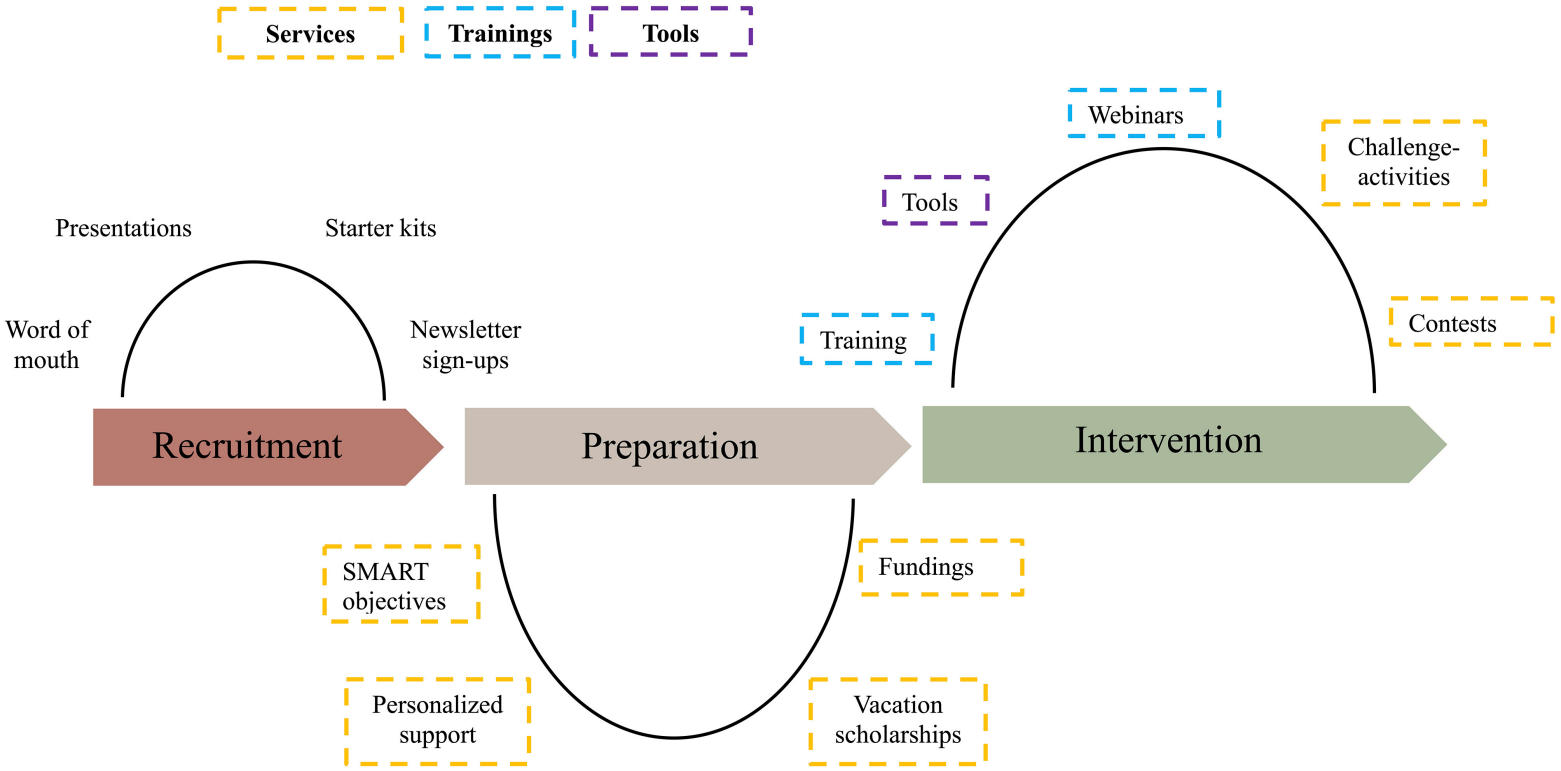
Power Up intervention program.

Power Up is a health promotion program developed and implemented by the Tremplin Santé Foundation, whose mission is to encourage the adoption and maintenance of healthy lifestyle behaviors among children and youth through summer camps. The program emphasizes fun, collaboration, and engagement to inspire participants to incorporate physical activity and healthy eating into their daily routines. To support camps in achieving these goals, Power Up provides a range of services, trainings, and tools tailored to facilitate the integration of healthy lifestyle habits (Figure 1). The program is powered by a multidisciplinary team of managers, dietitians, kinesiologists, and other professionals who collaborate to deliver resources and guidance, ensuring that camps are equipped to promote healthy lifestyle behaviors in an engaging and sustainable manner. Based on the Social Cognitive Theory (12), the program focuses on developing counselors' self-efficacy through educational activities, while also addressing interaction with the environment, rewarding positive behavior, and establishing role models. The implementation of Power Up does not follow a predetermined sequence, allowing camps to choose services, trainings, and tools according to their specific needs. New camps are recruited primarily through word of mouth and optional resources, such as presentations, starter kits, and newsletter sign-ups.

#### Services

Services such as personalized support, access to vacation scholarships, and funding are reserved for camps that have established SMART objectives for adopting healthy lifestyle habits before the summer begins. Personalized support is provided to camps that require assistance in achieving their healthy lifestyle objectives for the summer. This support can take various forms, such as help with implementing specific objectives, assistance in securing funding for materials, or providing guidance on how to engage the camp staff in promoting healthy lifestyle behaviors. Additionally, vacation scholarships are provided by Power Up to support camps in encouraging the participation of children, particularly those from low socio-economic status, in camp programs. This support helps ensure that financial barriers do not prevent these youth from benefiting from special activities that promote healthy lifestyles. The funding awarded is specifically intended to support projects that help achieve these objectives. Additionally, Power Up offers various services accessible to both camps already engaged with the program and new participants. To inspire and reward camps, Power Up organizes challenge-based activities and contests throughout the summer, focusing on themes such as physical activity, screen time reduction, healthy eating, and hydration. These contests are designed to recognize camps that excel in implementing challenge-based activities or initiate initiatives that align with their objectives of promoting healthy lifestyle habits (Table 1).

**Table 1:** Power Up program.

**Category**	**Activities**	**Description/Examples**
Services	Personalized support	Assistance in setting and achieving SMART objectives, securing funding, or engaging staff.
	Vacation scholarships	Providing financial aid to ensure children from low socio-economic backgrounds can participate.
	Fundings	Providing resources to camps, such as financial support for purchasing sports equipment or supplies for culinary activities.
	Challenge-based activities	Summer challenges on physical activity, screen time reduction, healthy eating, and hydration (Campaign Tchin Tchin Challenge).
	Contests	Contests to recognize camps that excel in implementing challenge-based activities or in promoting healthy lifestyle behaviors (Golden Legends Contest).
Trainings	Training for coordinators	Full-day training with guest speakers to enhance management skills and promote healthy habits.
	Training for counselors	One-hour initial training on key concepts, plus 30-min advanced workshops on specific topics.
	Webinars for camps	Thirty-minute video capsules to help implement challenges and activities throughout the summer.
Tools	To promote physical activity	• Free and Active Play • FUNdamentals to get moving!
	To promote sedentary behaviors	• Creation of a “pause your screen and enjoy” poster
	To promote healthy eating	• Lunchtime Icebreaker • Fun With Cooking
	To promote well-being	• Game cards—Let's take our time • Recharge yourself

#### Trainings

Training sessions are conducted directly by the Power Up team. For camp coordinators and managers, the program offers an engaging full-day training featuring expert guest speakers and interactive discussions to enhance their ability to manage counselors and promote healthy lifestyle habits. Similarly, counselors receive a 1-h training session on key concepts related to healthy lifestyle habits and guidance on using the program's tools, which are available on a dedicated website. In addition to the initial training, advanced 30-min sessions are available for counselors, covering topics like body image, camp meals, and avoiding stereotypes in activities, through interactive games and workshops. The program also offers webinars consisting of brief 30-min video capsules that assist camps in implementing challenges, contests, and other activities throughout the summer (Table 1).

#### Tools

The Power Up program offers more than 150 tools designed to promote healthy lifestyle habits, including physical activity, reducing sedentary behaviors, healthy eating, and wellbeing. These tools cover various activities, from brief 5-min sessions to extended activities lasting over 60 min, which can be conducted individually or in teams. Most activities require common materials typically found in all camps. For those requiring specific educational materials, Power Up provides these resources to any camp upon request, ensuring that all activities remain easily accessible. Each activity comes with an informational sheet that includes a description of the activity, required materials, objectives, and skills to be developed. These tools are specifically designed to enhance the daily camp experience, making it easier for counselors to integrate healthy lifestyle habits into their programming (Table 1).

### Evaluation based on a logic model

**Figure 2:**
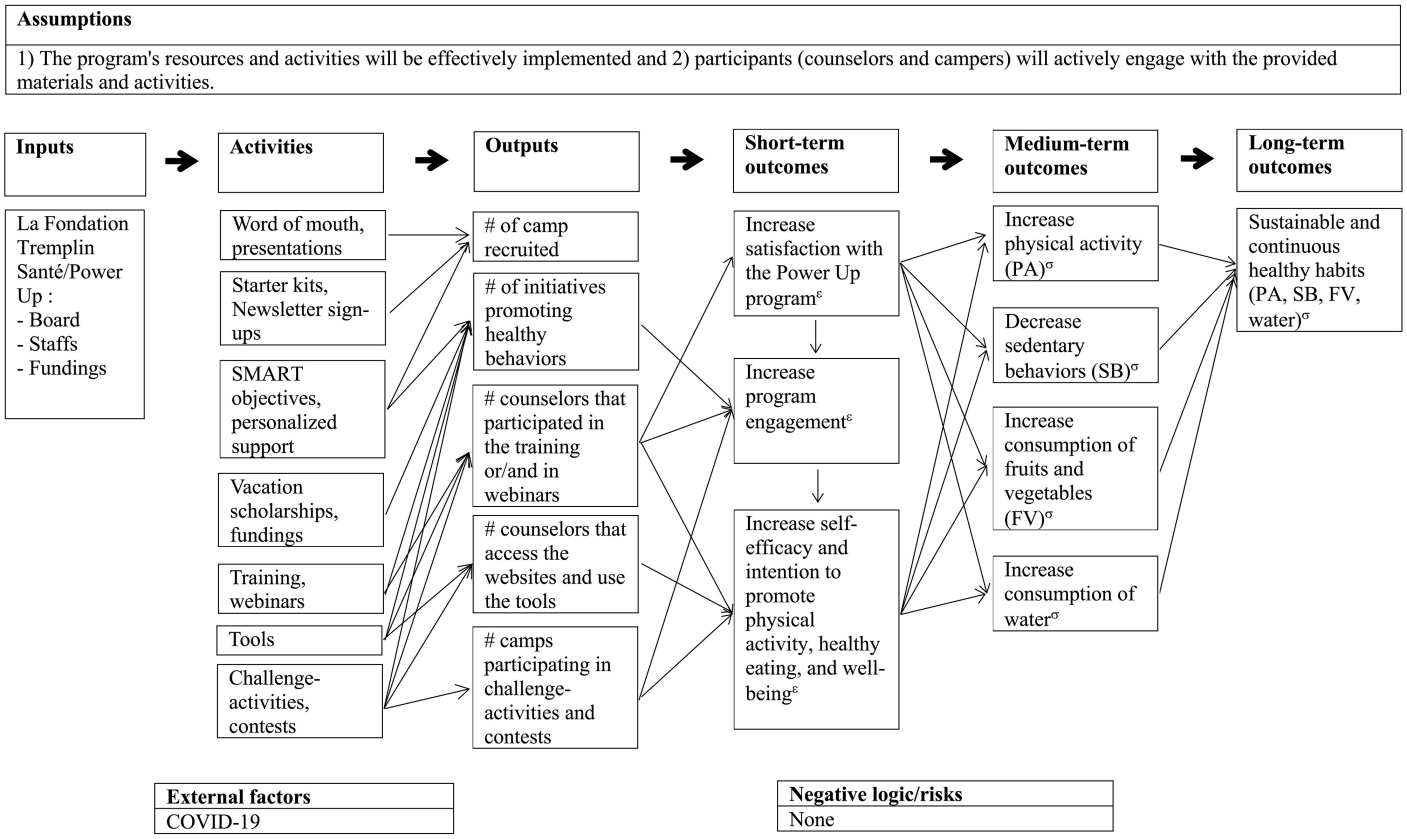
Evaluation logic model of Power Up: short-, medium-, and long-term results. ε, measured among counselors; σ, measured among campers and counselors.

The program components and the expected short-, medium-, and long-term results have been integrated into a logic model where arrows demonstrate the sequence of events and causal links from inputs, activities, and outputs to outcomes (Figure 2). The initiative relies on various resources, including an administration board that provides oversight and strategic direction, dedicated staff who implement and support the program, and financial resources to sustain the initiative. The Power Up program conducts several activities targeted at camps and counselors to create a healthy environment that encourages campers to engage in more physical activity, reduce sedentary behaviors, and adopt healthier eating habits. These activities lead to measurable outputs such as its promotion and reach including the number of camps recruited, the count of special initiatives promoting healthy behaviors within camps, the number of counselors who participate in training and webinars, the frequency of website and tool use during summer, and the number of camps involved in challenge-activities or contests. The anticipated short-term effects of the program include enhanced counselors' satisfaction with the Power Up program's services, trainings, and tools, along with greater engagement in the program. Counselors are also expected to develop stronger self-efficacy and intention to promote physical activity, healthy eating, and wellbeing. This satisfaction has the potential to influence medium-term outcomes, as counselors who are more satisfied with the program are likely to serve as positive role models for campers, reinforcing healthy behaviors. Counselors are also expected to develop stronger self-efficacy and intention to use the tools provided, which can further enhance their ability to promote physical activity, healthy eating, and wellbeing. In the medium term, the program aims to influence both counselors and campers by maintaining or increasing physical activity, maintaining or reducing sedentary behaviors, and maintaining or improving intake of fruits, vegetables, and water. The long-term goals of Power Up are to establish lasting healthy lifestyle habits, ultimately leading to improved overall health and wellbeing for both counselors and campers.

The logic model assumes that the program's services, trainings, and tools will be effectively implemented as planned and that counselors and administrators will actively engage with them. The COVID-19 pandemic is a significant external factor that could impact the program's implementation and outcomes, potentially affecting the number of participants and the activities within summer day camps. This logic model displays Power Up's program theory which is useful for both the planning and evaluation of Power Up's mission to promote healthy lifestyles.

### Data collection

This project will include both self-reported and direct observation data collection methods. The self-reported data will be gathered through questionnaires administered to counselors and campers. Data collection for campers is designed to be an enjoyable experience. The group will be divided into two sub-groups: while one half participates in fun physical activities, the other half will complete the questionnaire with the support of the research team. Afterward, the sub-groups will switch roles and the session will conclude with a physical activity game involving all the campers. Counselors will be invited to complete the questionnaire alone, either after work or during breaks, without the assistance of the research team. Counselors participating in the evaluation will first complete a healthy lifestyle questionnaire (HLQ-PRE) before attending the Power Up training. Following the training, they will fill out a training evaluation questionnaire (TEQ). At the end of the camps, counselors will complete the healthy lifestyle questionnaire again (HLQ-POST). Similarly, campers will complete the healthy lifestyle questionnaire during the first week of the camp (HLQ-PRE) and at the end of the summer (HLQ-POST).

**Figure 3:**
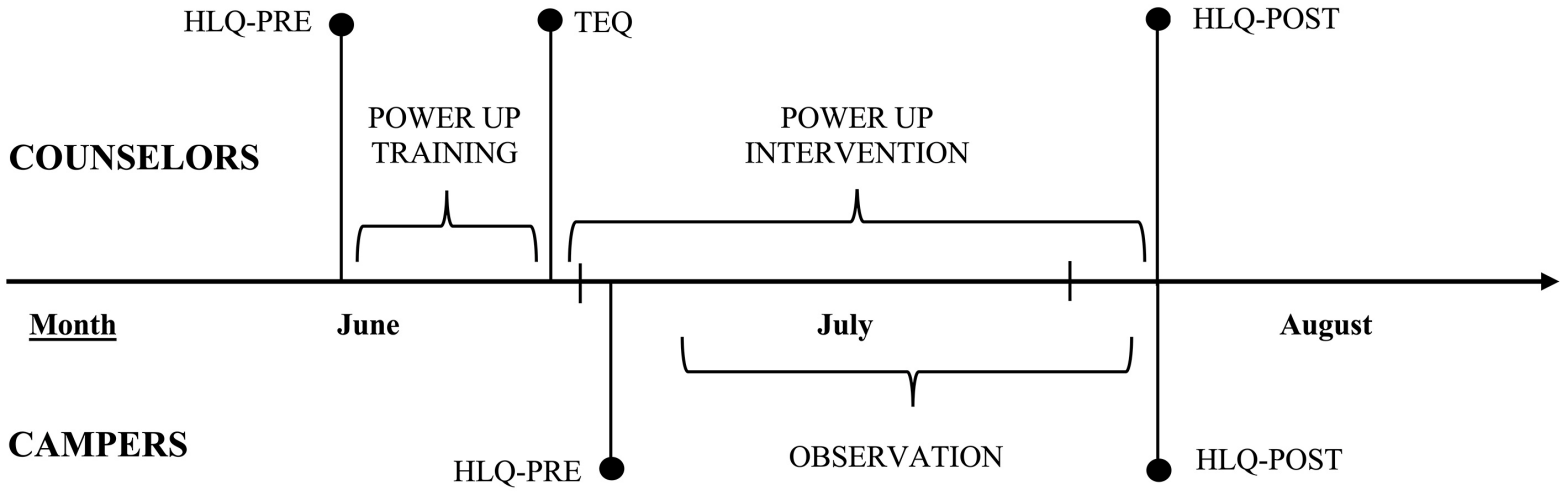
Implementation protocol and evaluation of Power Up. HLQ-PRE, Healthy Lifestyle Questionnaire completed before training (counselors) or at the beginning of the summer (campers); HLQ-POST, Healthy Lifestyle Questionnaire completed at the end of the summer (counselors and campers); TEQ, Training Evaluation Questionnaire completed after training (counselors).

Observations data will be collected throughout the summer using validated tools in Thirty-minute intervals over 2 days in each camp, with two observations during the first activity, two observations before lunch, one at lunchtime, and three observations before the afternoon snack (Figure 3). To ensure consistent and accurate data collection, the research staff underwent comprehensive training and data collection will be performed by two evaluators at the same time. This training included an explanatory video on using the assessment tools, a detailed mapping of the camps, and an in-depth explanation of each tool. Additionally, the training involved a full day of practical exercises to familiarize the staff with real-world application scenarios. Each researcher will also be paired with an experienced team member who had at least one summer of experience using the observation tools and working on the research project, providing ongoing guidance and support throughout the data collection period.

### Measurement tools

#### Self-reported

##### Healthy lifestyle questionnaire

The healthy lifestyle questionnaire was based on several existing questionnaires designed for youth. It includes items related to the evaluation of physical activity (e.g., number of active days in a week, participation in organized sports, and the number of practices/games per week) (13) along with interest (14), intention (15), perceived barriers and benefits (14, 16), perceived skills (16), and enjoyment related to physical activity (16). Additionally, this questionnaire measures sedentary behaviors (e.g., time spent in various sedentary activities such as TV, video games, and social media) (15), dietary habits (e.g., number of portions of various food consumed yesterday) (14, 15), and attitudes and intentions toward them (17–19).

The questionnaire also includes general questions about behaviors (i.e., the number of times they had breakfast, carried a water bottle with them, helped their parents with grocery shopping, helped their parents with cooking, and did gardening at home in the last week) (17), demographic questions (e.g., age, biological sex, gender, date of birth, educational level) (20), and questions about child poverty and deprivation aiming to provide an understanding of the perceived socio-economic context (e.g., a computer at home, traveling with the family outside of Canada in the last year, children aged 10 and older having separate rooms) (21).

The pre- and post-questionnaires for counselors and campers are slightly different. For instance, the pre-questionnaire for counselors asks about previous participation in Power Up training and work experience at the camp, while the camper version inquiries about the number of weeks spent at the camp, considering that data collection occurs during the first 2 weeks. In the post-questionnaire, counselors will be asked about the use and effectiveness of resources and support provided by their coordinator to promote healthy lifestyles, while campers will be asked about their overall satisfaction with the camp and whether counselors promoted healthy lifestyles through various activities.

##### Training evaluation questionnaire

The Power Up training evaluation questionnaire assesses several aspects, including counselors' satisfaction with the training, their self-efficacy, and their intention to promote physical activity, healthy eating, and wellbeing. It also evaluates whether the information and tools provided during the training are useful for counselors in planning activities with campers. Additionally, the questionnaire gathers insights on counselors' recall of key messages from the training, the activities they plan to implement during the summer, and the challenge-based activities and contests they intend to participate in. It collects data on the training context as well, such as whether it was conducted in-person at the camp or online with other counselors or coordinators from the same camp.

#### Observation tools

##### System for observing play and leisure activity in youth

The System for Observing Play and Leisure Activity in Youth (SOPLAY) evaluates campers' physical activity and the surrounding environmental contexts (22, 23). Following a specific protocol, this tool allows the research team to map camps into multiple zones, where two team members then observe campers by conducting scans in each zone several times a day. SOPLAY provides data on the number of boys and girls and their activity levels within a particular environment. During each observation, the physical activity of each camper is categorized as sedentary/inactivity (lying, sitting, or standing), walking, or very active. Separate scans are performed for boys and girls, and information is simultaneously recorded about the time of day, temperature, and details of the physical environment (e.g., area accessibility, usability, supervision, type of organized activities, and availability of equipment). Additionally, questions related to the food environment have been integrated into the tool to better understand campers' habits (e.g., the presence of water fountains, refrigerators, microwaves, informational posters, vending machines, and gardens).

##### System for observing staff promotion of activity and nutrition

The System for Observing Staff Promotion of Activity and Nutrition (SOSPAN) evaluates camp staff actions and contextual factors related to promoting physical activity and healthy eating through structured observations, following the same procedure as the SOPLAY (24). The SOSPAN contains three main categories: (1) management of physical activity (e.g., staff giving other instructions, staff disciplining children, idle time, children standing in line for their turn, elimination game) and healthy eating (e.g., nutrition promotion, nutritional education, food safety) by staff; (2) staff behavior toward physical activity (e.g., leading physical activities, engaging in physical activity, supervising) and healthy eating (e.g., staff consuming foods other than fruits or vegetables, staff consuming fruits and vegetables, staff drinking water); and (3) contextual factors including the number of counselors in the observed zone.

### Data analysis

Camper data will be collected using paper questionnaires and entered via Qualtrics. Counselors will complete their questionnaires online directly via Qualtrics. To ensure data accuracy, all camper questionnaires and observational data will undergo double entry. Logistical and resource constraints complicate the inclusion of a control group; thus, the focus remains on assessing the program's real-world implementation and effectiveness. To document the effect of the program, an implementation score will be created to assess the extent of the Power Up intervention program's application in the camps. This score will be derived from several factors: the number of years participating in the program (i.e., 0 for the first year, 1 for the second year, 2 for the third year, and 3 for the fourth year and beyond), the presence of projects promoting healthy habits (i.e., 0 for none, 1 for present), engagement in challenge-based activities (i.e., 0 for no challenge, 1 for one challenge, 2 for two challenges, 3 for three challenges, and 4 for four challenges), participation in contests (i.e., 0 for no contest, 1 for one contest, 2 for two contests, 3 for three contests, 4 for four contests, and 5 for five contests), and the reported use of tools by counselors (i.e., percentage of counselors reporting the use of at least one tool during the summer). Based on the median of the total implementation score, camps will be categorized into two groups: high implementation and low implementation. This categorization will allow for comparative analysis of the program's effect on counselors and campers. Observational measurements will be used to understand the physical activity and food environments within the camps and will be analyzed as indicators of these environments.

Data will be analyzed using SAS software (version 9.4; SAS institute Inc., Cary, NC, USA). Extreme values will be excluded using a method based on three standard deviations (25). Descriptive analysis and MIXED linear models for repeated measures will be carried out to evaluate the effects of time, group (implementation level), and their interaction on behaviors (i.e., physical activity, sedentary behaviors, and healthy eating) as well as on the determinants of these behaviors (e.g., intention, obstacles and facilitators, attitude, perceived control). Reliability for SOPLAY and SOSPAN will be assessed using Cohen's Kappa and Intraclass Correlation Coefficients (ICCs) where appropriate. Tukey's *post hoc* test will be used to identify differences when a group x time interaction is observed. Statistical significance will be set at *p* < 0.05.

## Discussion

This paper describes the protocol of a quasi-experimental study aiming to assess the effectiveness of the Power Up intervention. Power Up has developed a comprehensive range of services, trainings, and tools specifically designed to help camps promote healthy lifestyle habits among counselors and campers, based on the Social Cognitive Theory (12). Implementing Power Up activities allows camps to choose resources according to their specific needs throughout the summer. Overall, Power Up endeavors to create an environment conducive to healthy living and acknowledges camps for promoting healthy lifestyle habits among campers. The expected short-term effects of the Power Up program include enhanced satisfaction with the program, greater engagement in the program, and increased self-efficacy and intention among counselors to promote physical activity and healthy eating. The medium-term effects involve maintaining or changing counselors' and campers' behaviors, including maintaining or increasing physical activity, maintaining or reducing sedentary behaviors, and maintaining or enhancing fruit, vegetable, and water intake. The long-term goals aim for lasting and consistent healthy lifestyle habits and improved health among all counselors and campers.

The study has several notable strengths that support its validity. The 3-year duration of the data collection and its evaluation across two Canadian provinces accounts for regional disparities and camp-specific characteristics and will provide a comprehensive overview of the findings. Also, following the same participants at the beginning and end of the summer will allow for a complete understanding of the intervention's effects and the large sample size of campers aged 8 to 12 and counselors aged 15 to 21 will facilitate a thorough exploration of the intervention's effect in different age groups and perspectives. Another strength of this study is the use of personalized questionnaires incorporating validated questions from similar settings and validated observation tools, ensuring the reliability of the data collected. Finally, the meticulous process of data collection and double data entry will guarantee the accuracy and consistency of the data. These combined strengths enhance the credibility and robustness of the study for assessing the effectiveness of the Power Up intervention in the summer camp setting.

The study also has some limitations that are worth mentioning. The study lacks a real control group for practical, ethical and feasibility reasons. However, an implementation score allowing comparisons of groups with different levels of program utilization will be used to address this limitation. Another limitation is the absence of an objective measure of physical activity such as accelerometry, primarily due to challenges in obtaining parental consent for pedometer assessments, time constraints, difficulty in capturing certain activities, and the potential loss of devices. The study will provide valuable insights into the effectiveness of the Power Up program, but these limitations should be considered when interpreting the findings.

## Conclusions

The Power Up evaluation provides a valuable opportunity to improve the program and better target its implementation among camps and counselors. Furthermore, this evaluation will directly measure key factors influencing the adoption of healthy lifestyle habits among counselors and campers as well as supportive environments. This project stands out for its emphasis on physical activity, sedentary behaviors, and healthy eating in the context of summer day camps, an under-explored area of research. Additionally, using observation tools adapted to the extracurricular context will provide valuable information on camp environments to improve the lifestyle habits of young Canadians.
